# The role of TMS in the clinical trajectory of patients with resistant Obsessive Compulsive Disorder Systematic Review

**DOI:** 10.1192/j.eurpsy.2025.699

**Published:** 2025-08-26

**Authors:** U. Anyeji, E. O. Molokwu, O. Alli-Balogun

**Affiliations:** 1 Adult Psychiatry, BronxCare Health System, Bronx, United States; 2 Psychiatry, Windsor, Canon, Saint Kitts and Nevis

## Abstract

**Introduction:**

- Obsessive- Compulsive Disorder (OCD) is a neuropsychiatric illness affecting 2-3% of the United States population during their lifetime. It is a highly prevalent chronic disorder, often refractory to treatment, an understanding of the pathophysiology of OCD is crucial to optimize treatment. The most common comorbid diagnosis is major depressive disorder (MDD). In 2018, the US Food and Drug Administration cleared the treatment of resistant OCD with high frequency deep repetitive transcranial magnetic stimulation (dTMS) over the dorsomedial prefrontal and anterior cingulate cortices (DMPFC-ACC) with the H7 coil based on the efficacious results of a multi-center study. Compare to H7 coil, H1 coil which is approved for the treatment of resistant MDD, targets and stimulate the left prefrontal cortex more than the medial and right prefrontal cortices.
- Carmi L, Tendler A, Rodrigues da Silva D,

**Objectives:**

To assess the effect of TMS in patients with refractory OCD

**Methods:**

We conducted literature review search on treatment- specific for OCD on four databases, i.e google scholar, PubMed, PsycINFO and Mount Sinai’s Levy Library.

**Results:**

The meta-analysis combined the results of individual randomized controlled trials (RCTs) to evaluate the effectiveness of repetitive transcranial magnetic stimulations (rTMS) on obsessive-compulsive disorder (OCD). It showed that rTMS were moderately effective in reducing OCD symptom severity (studies indicated a medium-sized effect, Hedges’ g = 0.59-0.65) and a threefold augmentation of treatment effects as compared with sham conditions. Studies also reported a marked heterogeneity in treatment response, which appeared to be particularly seen in patients with comorbid depression. Clinical improvement in depression was associated with greater reductions in OCD symptoms.

Certain rTMS protocols, including low-frequency stimulation (LF-rTMS) over the dorsolateral prefrontal cortex (DLPFC) and supplementary motor area (SMA), consistently showed more effects. Longer session durations and targeted stimulation of non-DLPFC regions, such as the orbitofrontal cortex and pre-SMA, were also associated with more positive outcomes.

But there are limitations - These studies were small, heterogeneous, and prone to publication bias, and few studies reported serious side effects, though some protocols especially those using high-frequency stimulation yielded higher rates of adverse effects.

**Conclusions:**

To conclude, rTMS use as a new therapy for treatment-resistant OCD, particularly OCD comorbid with depression, is promising. However, due to study design inconsistencies and limited statistical power in individual trials, the quality of evidence is currently low. Studies using this approach need further improvement and more evidence to refine stimulation parameters better, increase our understanding of the underlying mechanisms, and confirm sustained efficacy.

**Disclosure of Interest:**

None Declared

